# Effects of portal vein resection and hepatic artery resection on long-term survival in Klatskin tumor: a meta-analysis

**DOI:** 10.1186/s12957-022-02692-1

**Published:** 2022-07-12

**Authors:** Yun Song, Yujie Zhang, Zhijie Zhen, Zhaohui Huang

**Affiliations:** 1 Wuxi School of Medicine, Jiangnan University, Wuxi, Jiangsu People’s Republic of China; 2 Wuxi Cancer Institute, Affiliated Hospital of Jiangnan University, 200 Huihe Road, Wuxi, 214062 Jiangsu People’s Republic of China

**Keywords:** Portal vein resection, Hepatic artery, Klatskin tumor, Survival, Meta-analysis

## Abstract

**Background:**

Surgical treatment is currently the only way to achieve the clinical cure for Klatskin tumor. However, whether combined vascular resection should be combined during surgeries is still controversial. The aim of this article was to analyze the effect of portal vein resection (PVR) and hepatic artery resection (HAR) on the long-term survival after surgery for Klatskin tumor.

**Methods:**

Articles about Klatskin tumor with PVR and HAR, which were published from 2000 to 2020, were searched using PubMed, Embase, and EBSCO. HR with a 95% CI of overall survival, recurrence-free survival, disease-free survival, 3- and 5-year survival rate, and median survival time were reported to evaluate prognosis.

**Results:**

A total of 17 articles were included. The total case number of these studies was 3150 (685 in the PVR group, 345 in the HAR group, and 2120 in the control group). Survival analyses showed that both vascular resection types were poor prognostic factors (PVR: HR = 1.50, 95% CI = 1.24–1.81, *P* < 0.001; HAR: HR = 1.68, 95% CI = 1.26–2.24, *P* < 0.001; the pooled effect size of the two groups: HR = 1.55, 95% CI = 1.32–1.82, *P* < 0.001). In general, the analyses of 3- and 5-year survival and median survival time showed that both vascular resection types tended to be poor prognostic factors, but most of recent researches showed that the PVR did not lead to a poor prognosis.

**Conclusion:**

PVR should be used when necessary to achieve R0 resection of Klatskin tumor and improve the long-term survival of patients. Whether HAR should be performed or not is still need to be evaluated.

## Introduction

Cholangiocarcinoma ranks the second most common primary liver cancer, while Klatskin tumor (also known as perihilar cholangiocarcinoma or hilar cholangiocarcinoma) accounts for 50–60% of cholangiocarcinoma [1]. Klatskin tumor is a malignant disease with a poor prognosis, and the surgical resection is still the only way to achieve the clinical cure for this cancer type [2, 3]. The first resection of Klatskin tumor was reported in 1954 [4]. The resection extent of Klatskin tumor has been continuously extended from the resection of bile duct with affected liver parenchyma at the early stage. The hilar is close to the portal vein and hepatic artery, which are easily invaded by cancer cells.

Therefore, vascular resection and reconstruction is commonly applied to Klatskin tumor to reduce the recurrence rate and to obtain a clear margin. Some researchers advocated the hepatectomy with total portal vein resection (PVR) to improve the curative resection rate of Klatskin tumor [5, 6], while other researchers recommended hepatic artery resection (HAR) in specific cases [7, 8]. It is still controversial whether combined vascular resection and reconstruction improves the survival of patients and whether both PVR and HAR should be performed. Several studies have shown that the PVR group has a worse long-term prognosis than the non-vascular resection group [9–12], while some other studies hold different views [13, 14], and a similar situation is seen in studies about the value of HAR in Klatskin tumor [10, 13, 15].

This meta-analysis aimed to clarify the effects of combined vascular resection and reconstruction on the long-term survival of Klatskin tumor patients.

## Materials and methods

The present meta-analysis was performed in accordance with the PRISMA guidelines [16], which was registered on the INPLASY platform with the registration number INSPLASY202230042 (10.37766/inplasy2022.3.0042). All parts of the materials and methods can also be found in this registered protocol by us (10.37766/inplasy2022.3.0042) [17].

### Search strategy

Relevant articles were searched using the following electronic databases: Embase, EBSCO, and PubMed, and the keywords included hilar cholangiocarcinoma, Klatskin tumor, hilar bile duct cancer, hepatic artery resection, vascular resection, and portal vein resection. The full search strategy can be found below:

- PubMed: the formula was ((Hilar Cholangiocarcinoma) OR (Hilar Bile Duct Cancer)) OR (Klatskin Tumor)) AND ((Vascular Resection) OR (Hepatic Artery Resection) OR (Portal Vein Resection)); the search period was set from January 2000 to December 2020.
- Embase: the formula was (vascular AND resection OR (hepatic AND artery AND resection) OR (portal AND vein AND resection)) AND (hilar AND cholangiocarcinoma OR (hilar AND bile AND duct AND cancer) OR (klatskin AND tumor)) AND [2000–2020]/py.
- EBSCO: the formula was (Hilar Cholangiocarcinoma OR Hilar Bile Duct Cancer OR Klatskin Tumor) AND (Vascular Resection OR Hepatic Artery Resection OR Portal Vein Resection); the expanders selected were “Apply related words”, “Also search within the full text of the articles”, and “Apply equivalent subjects”; the limiters set was “Published Date: 20000101-20201231”.

In addition, reference lists of all these retrieved articles were also manually checked and searched to find additional studies missed by the aforementioned search.

### Selection criteria [15]

Articles indicating a correlation between prognosis and the presence or absence of PVR or HAR for Klatskin tumor in the above potentially relevant studies. The range of included papers was from 2000 to 2020 retrieved in the database.

### Inclusion criteria [15]

1. English papers and human studies.
2. Papers on surgeries combined with vascular resection for Klatskin tumor.
3. The hazard ratios (HR) and 95% confidence interval (CI) of patients between the vascular resection group and non-vascular resection group (control group) or the prognostic or the survival curve that can be used to extract the data were presented in the paper.
4. Survival types included were overall survival (OS), recurrence-free survival (RFS), or disease-free survival (DFS).

### Exclusion criteria [15]

1. Articles on palliative surgery or without a control group.
2. Articles not identifying the type of vascular resection or the type of resection that did not belong to the PVR or HAR when describing the prognostics of patients.
3. Articles without complete data or graphs required.
4. Individual case report or studies with less than 10 eligible cases included.
5. Repeated articles.
6. The study with the largest sample size was selected when articles with the same series of cases were reported repeatedly by the same author.

### Data extraction and study quality evaluation [15]

The following data were extracted from the eligible studies: first author, country, date of publication, type of resection, total case number, the case number in the vascular resection group or the control group, 3- and 5-year survival rate, median survival time, and the HR with 95% CI. Emails were sent to the original corresponding authors of included studies for confirmation of uncertain data. The final data extraction results were presented in tabular form. The Newcastle-Ottawa Scale (NOS) 9 scoring standard was applied to evaluate the quality of all these included studies indepentently by two authors. A total of 9 assessment indicators were used to evaluate “selection, comparability, and exposure”. These indicators were scored from 0 to 9 points, and those studies with 5–9 points were included in this meta-analysis.

### Statistical analyses

HR with the 95% CI of patient survival (OS, DFS, or RFS) and the median survival time were used to assess the association between the status of vascular resection and the survival in Klatskin tumor. An observed HR > 1 indicates a worse prognostic significance for the corresponding vascular resection group compared with the control group. In contrast, HR < 1 indicates a better prognostic significance of the vascular resection. In addition, the relative risk (RR) was calculated for the 3- or 5-year survival rate with the similar method as HR.

Statistical analyses were performed using StataSE 15.1 software (Stata Corporation, USA). The heterogeneity of the effect sizes was assessed by *I*^2^ statistics. High heterogeneity was considered to be present if the *I*^2^ > 50%. The Random-effect model was used in the presence of significant heterogeneity; otherwise, the fixed-effect model was used. Meta-regression and subgroup analyses were used to assess possible factors leading to heterogeneity. Begg’s and Egger’s test was used to evaluate publication bias which was considered to exist when a *P* < 0.10. Sensitivity analyses were also used to assess publication bias.

## Results

### Study selection

**Fig. 1:**
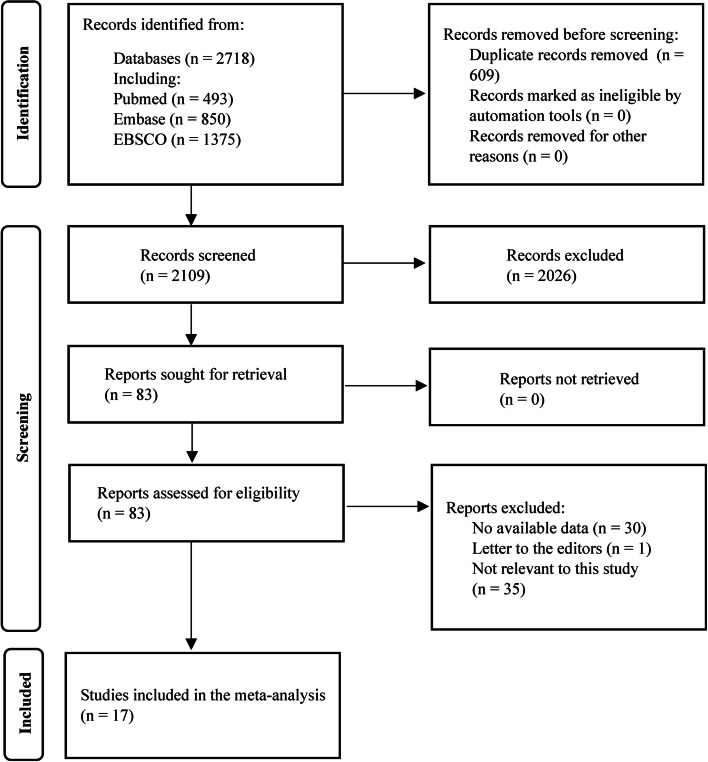
Flow chart showing the study selection process (in accordance with the PRISMA flow diagram)

A total of 2718 relevant articles were initially retrieved and independently screened by two authors. By removing duplicate reports, reading titles and abstracts, and excluding articles unrelated to the study, 83 studies were screened out. After assessing the full text of these 83 studies, 17 studies were finally included according to the inclusion and exclusion criteria [7, 9–15, 18–26], which were all retrospective studies (Fig. 1). All studies were identified as eligible for inclusion in the study by the NOS score (Table 1). The cumulative sample size was 3150, including 1030 patients in the vascular resection group (685 patients with PVR and 345 patients with HAR) and 2120 in the control group. The basic characteristics of each study and HR with 95% CI were shown in Table 2. The 3- and 5-year survival rate and median survival time of the studies included in the meta-analysis were shown in Table 3.

**Table 1:** The basic characteristics of the studies included in the meta-analysis

Author	Country	Year	Case timing	*n* (Control group)	*n* (PVR)	*n* (HAR)	Quality score
Nimura et al. [18]	Japan	2000	1977–1997	99	43	–	6
Muñoz et al. [19]	USA	2002	1990–2001	18	10	–	7
Ebata et al. [9]	Japan	2003	1979–2000	98	47	–	7
Miyazaki et al. [10]	Japan	2007	1981–2004	118	34	9	7
Igami et al. [11]	Japan	2010	2001–2008	176	69	53	6
Hemming et al. [20]	USA	2011	1990–2010	53	42	–	6
de Jong et al. [21]	Multiple countries^a^	2012	1984–2010	173	51	–	7
Tamoto et al. [14]	Japan	2014	2005–2009	13	36	–	7
Yu et al. [12]	China	2014	1998–2010	174	25	47	8
Hoffmann et al. [22]	Germany	2015	2001–2012	39	21	–	7
Wang et al. [15]	China	2015	2005–2012	114	16	24	7
Peng et al. [7]	China	2016	2005–2012	35	–	26	6
Nakanishi et al. [23]	Japan	2016	1998–2015	74	94	–	5
Noji et al. [24]	Japan	2016	2000–2015	181	–	28	5
Dumitraşcu et al. [25]	Romania	2017	1996–2014	102	21	–	6
Schimizzi et al. [13]	USA	2018	1998–2015	169	19	12	5
Mizuno et al. [26]	Japan	2020	2001–2018	484	157	146	7

^a^Including USA, Portugal, Italy, and Switzerland; – represents missing data

**Table 2:** Original data and HR with 95% CI of the studies included in the meta-analysis

Study	HR	95%CI	Resection type	Survival type
Nimura et al. [18]	1.9	[1.52, 3.52]	PVR	OS
Muñoz et al. [19]	2.39	[0.55, 10.4]	PVR	OS
Ebata et al. [9]	2.5	[1.15, 5.42]	PVR	OS
Miyazaki et al. [10]	1.59	[0.83, 3.06]	PVR	OS
	2.92	[1.5, 5.67]	HAR	OS
Igami et al. [11]	1.83	[0.78, 4.27]	PVR	OS
	1.79	[0.76, 4.17]	HAR	OS
Hemming et al. [20]	1.05	[0.37, 3]	PVR	OS
de Jong et al. [21]	1.22	[0.57, 2.63]	PVR	OS
Tamoto et al. [14]	0.38	[0.1, 1.41]	PVR	OS
	0.3	[0.1, 0.87]	PVR	RFS
Yu et al. [12]	2.29	[1.09, 4.8]	PVR	OS
Hoffmann et al. [22]	0.64	[0.29, 1.41]	PVR	OS
	0.76	[0.35, 1.62]	PVR	DFS
Wang et al. [15]	1.61	[0.75, 3.43]	PVR	OS
	1.37	[0.65, 2.89]	HAR	OS
Peng et al. [7]	1.42	[0.63, 3.17]	HAR	OS
Nakanishi et al. [23]	1.34	[0.65, 2.77]	PVR	OS
Noji et al. [24]	1.96	[0.85, 4.57]	HAR	OS
Dumitraşcu et al. [25]	1.43	[0.68, 3.00]	PVR	OS
Schimizzi et al. [13]	0.9	[0.5, 2.2]	PVR	OS
	1	[0.5, 2.2]	HAR	OS
	1.7	[0.8, 3.3]	PVR	RFS
	0.6	[0.3, 1.3]	HAR	RFS
Mizuno et al. [26]	1.78	[0.86, 3.67]	PVR	OS
	1.7	[0.84, 3.46]	HAR	OS

**Table 3:** 3- and 5-year survival rate and median survival time of the studies included in the meta-analysis

Study	3-year survival rate (%)			5-year survival rate (%)			Median survival time (month)		
	Control	PVR	HAR	Control	PVR	HAR	Control	PVR	HAR
Nimura et al. [18]	40.4	18.6	–	27.3	4.7	–	29.0	13.4	–
Muñoz et al. [19]	44.4	20.0	–	38.9	20.0	–	48.0	25.0	–
Ebata et al. [9]	54.1	25.5	–	36.7	10.6	–	37.4	17.4	–
Miyazaki et al. [10]	39.8	17.6	11.1	29.7	14.7	0	24.0	11.2	7.0
Igami et al. [11]	56.8	37.7	37.7	51.1	33.3	22.6	64.1	22.1	19.9
Hemming et al. [20]	52.8	54.8	–	37.7	38.1	–	37.7	49.7	–
de Jong et al. [21]	37.6	37.3	–	22.0	29.4	–	22.9	18.8	–
Tamoto et al. [14]	53.8	66.7	–	53.8	58.3	–	20.5	20.5	–
Yu et al. [12]	27.6	20.0	19.1	21.8	0.0	6.4	–	–	–
Hoffmann et al. [22]	41.0	28.6	–	17.9	19.0	–	28.1	32.3	–
Wang et al. [15]	46.5	37.5	25.0	36.0	25.0	25	32.0	20	26.0
Peng et al. [7]	51.4	–	42.3	37.1	–	30.8	49.0	–	23.0
Nakanishi et al. [23]	45.9	45.7	–	21.6	25.5	–	51.4	41	–
Noji et al. [24]	53.6	–	35.7	26.5	–	17.9	47.1	–	27.7
Dumitraşcu et al. [25]	42	28	–	43	26	–	26	34	–
Schimizzi et al. [13]	33.3	26.3	50.0	–	–	–	21.0	24	45.0
Mizuno et al. [26]	62.9	38.2	49.2	50.1	24.6	29.5	60	29	34

Control, PVR, and HAR are the groups to which the data below belong; – represents missing data

### Outcomes

#### OS analyses

**Fig. 2:**
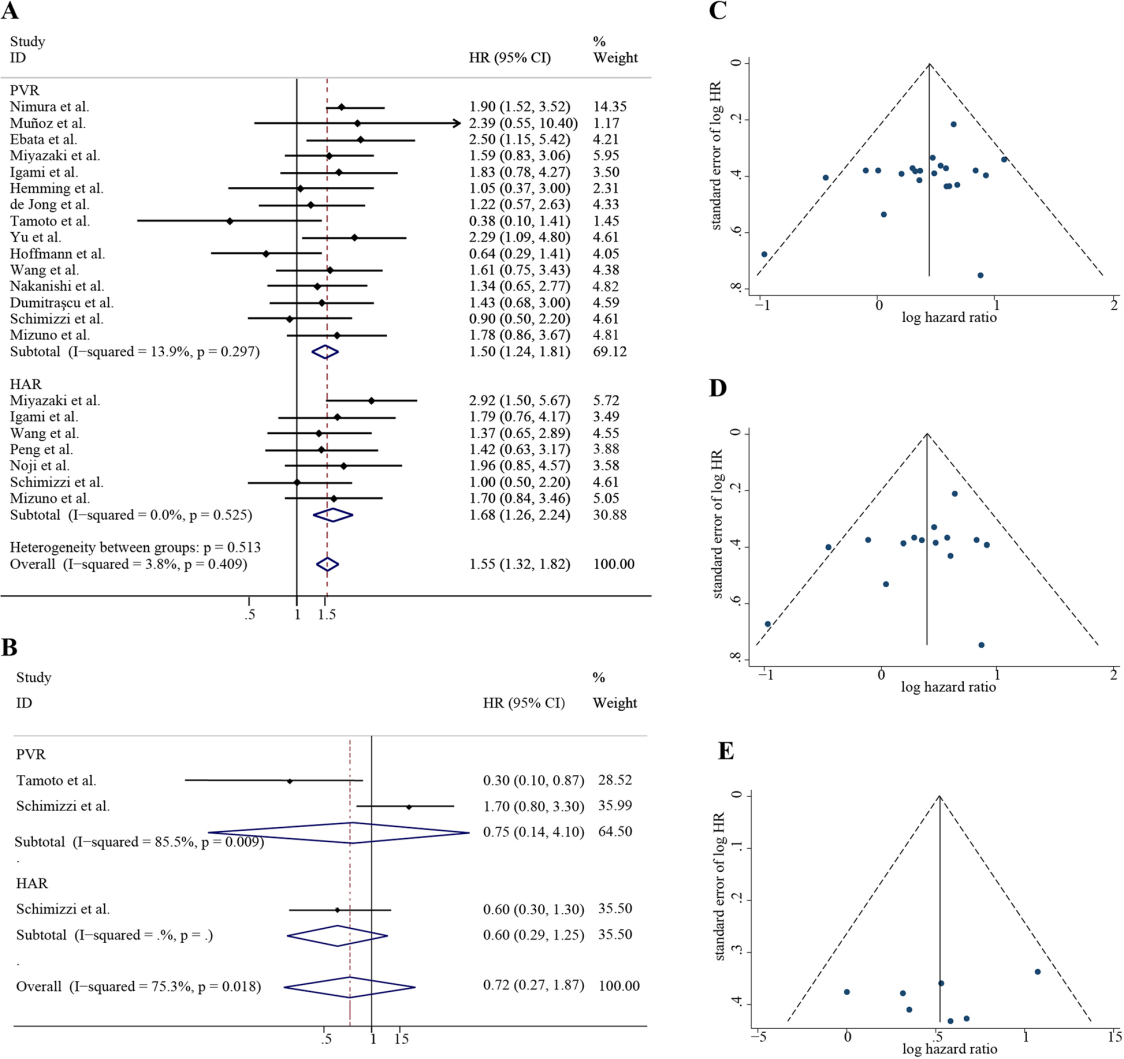
The analyses of OS, RFS, and DFS with the indictor of HR with 95% Cl. **A** The effects of PVR and HAR on the OS of hilar cholangiocarcinoma patients. **B** The effects of PVR and HAR on RFS. **C** The funnel plot of OS for vascular resection (Including PVR and HAR). **D** The funnel plot of OS for PVR. **E** The funnel plot of OS for HAR

The heterogeneity of both PVR and HAR was not significant (*I*^2^ = 13.9%, *P* = 0.297; *I*^2^ = 0.0%, *P* = 0.525) (Fig. 2A), and significant differences in OS existed between PVR or HAR group and their corresponding control group (PVR: HR = 1.50, 95% CI = 1.24–1.81, *P* < 0.001; HAR: HR = 1.68, 95% CI = 1.26–2.24, *P* < 0.001). Besides, significant difference also existed in the pooled effect size of the two groups (HR = 1.55, 95% CI = 1.32–1.82, *P* < 0.001) (Fig. 2A).

**Fig. 3:**
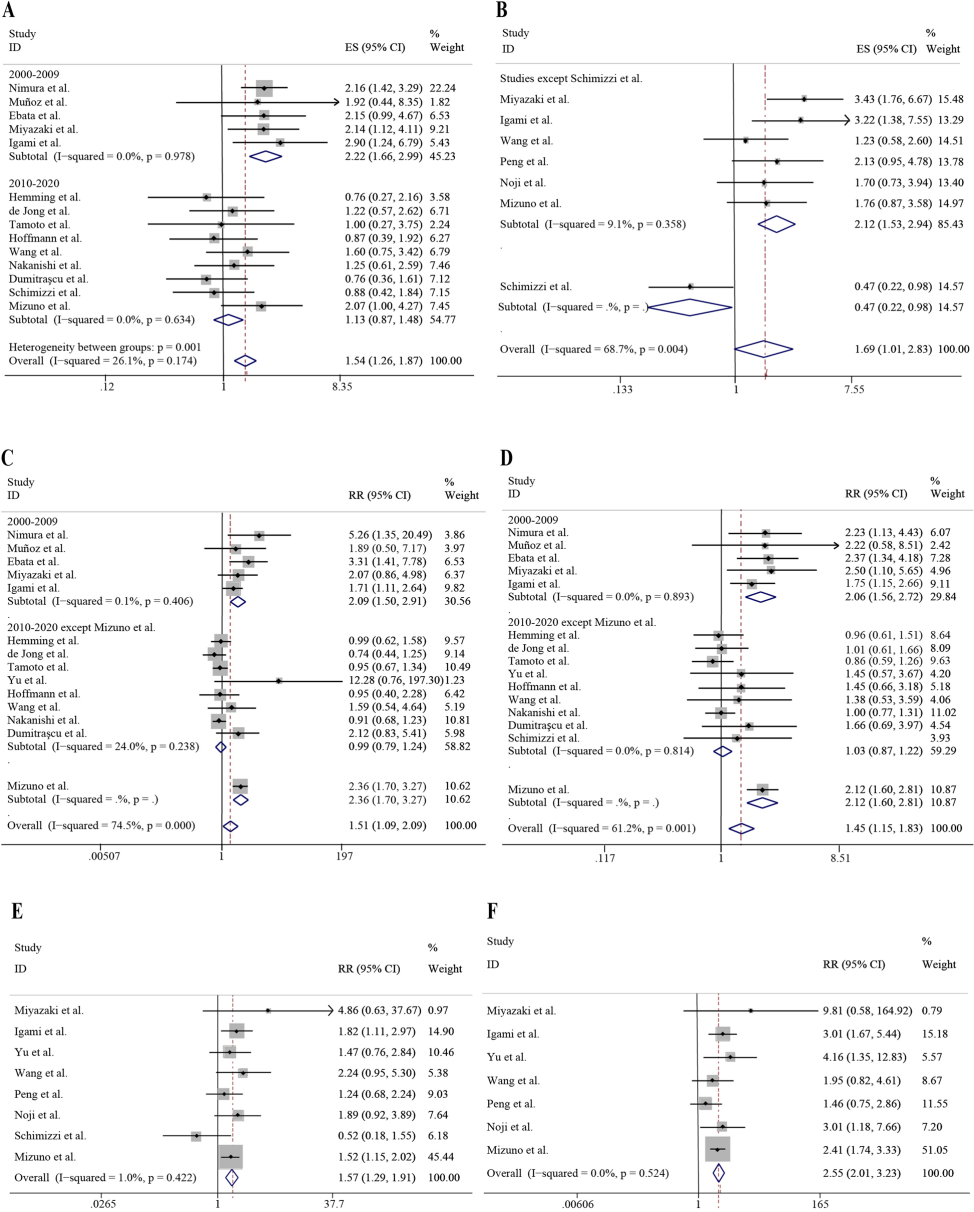
The analyses of 3- and 5-year survival rate, and median survival time. **A** The analysis on the RR of PVR by the 3-year survival rate. **B** The analysis on the RR of PVR by the 5-year survival rate. **C** The analysis on the RR of HAR by the 3-year survival rate. **D** The analysis on the RR of HAR by the 5-year survival rate. **E** The effects of PVR on the median survival time. **F** The effects of HAR on the median survival time

The median survival time data were obtained in 14 studies, and the meta-analysis in these studies showed a statistically significant difference in median survival time (HR = 1.54, 95% CI = 1.26–1.87, *P* < 0.001). Although the results were not highly heterogeneous (*I*^2^ = 26.1%), subgroup analyses were done to take into account the effect of the different study periods. Heterogeneity between the two subgroups of studies was further reduced (*I*^2^ = 0.0%; *I*^2^ = 0.0%), with the results suggesting a statistical difference in median survival time between the PVR group and the control group for studies prior to 2010, and no statistical difference for studies after 2010 (HR = 2.22, 95% CI = 1.66–2.99, *P* < 0.001; HR = 1.13, 95% CI = 0.87–1.48, *P* = 0.357) (Fig. 3E).

Median survival time was compared between the HAR group and the control group in 5 studies, and no statistical difference was observed. However, we noticed that significant heterogeneity exists among these studies (*I*^2^ = 68.7%; *P* = 0.004). Subgroup analyses suggested that the heterogeneity was significantly lower after excluding Schimizzi’s study and that the median survival time in the HAR group was significantly decreased compared with the control group (*I*^2^ = 9.1%; *P* = 0.358), while Schimizzi’s study showed the opposite trend (*P* = 0.044) (Fig. 3F).

### RFS and DFS analyses

The significant heterogeneity existed in RFS (*I*^2^ = 75.3%, random-effect model), and the heterogeneity of PVR was even higher (*I*^2^ = 85.5%) (Fig. 2B). Besides, because only one study contained the DFS data and the HAR group of RFS, the heterogeneity could not be evaluated in these two groups. In addition, no significant difference in RFS and DFS was observed between patients with or without vascular resection (*P* = 0.495; *P* = 0.483).

### Meta-regression analysis

Meta-regression analysis was performed with “study publication period” and “country” as independent variables, and the publication period was bounded by 2010, which showed that the publication period of the studies was the source of heterogeneity (*P* < 0.05), whereas “country” was not the source of heterogeneity (*P* > 0.05).

### The analyses of 3- and 5-year survival rate

To further evaluate the effects of vascular resection on patient survival, additional survival analyses were performed using 3- and 5-year survival index. A total of 15 studies compared the 3-year survival rates of the PVR group with the control group. The results overall suggested that PVR was a poor prognostic factor (RR = 1.45, 95% CI = 1.15–1.83, *P* = 0.001), and there was also relatively significant heterogeneity among these studies (*I*^2^ = 61.2%). Subgroup analyses showed that the main source of heterogeneity was the study period and Mizuno’s study. RR was statistically significant in studies prior to 2010 but not obvious in studies after 2010 after excluding Mizuno’s study (RR = 2.06, 95% CI = 1.56–2.72, *P* < 0.001; RR = 1.03, 95% CI = 0.87–1.22, *P* = 0.696). Heterogeneity was reduced evidently in both subgroups (*I*^2^ = 0.0%; *I*^2^ = 0.0%). The RR of Mizuno’s study was similar to that of studies prior to 2010 (RR = 2.12, 95% CI = 1.60–2.81, *P* < 0.001) (Fig. 3A).

In addition, similar results were also observed for the 5-year survival analyses of PVR. A poorer 5-year survival was also observed in the PVR group compared with the control group in 14 studies (RR = 1.51, 95% CI = 1.09–2.09, *P* = 0.013) (*I*^2^ = 74.5%). Source of heterogeneity in the 5-year survival analyses among studies was the same as the 3-year survival analyses. Subgroup analyses showed that RR was statistically significant in studies prior to 2010, but not in studies after 2010 when excluding Mizuno’s study (RR = 2.09, 95% CI = 1.50–2.91, *P* < 0.001; RR = 0.99, 95% CI = 0.79–1.24, *P* = 0.937). The RR of Mizuno’s study was statistically significant (RR = 2.36, 95% CI = 1.70–3.27, *P* < 0.001) (Fig. 3B).

Moreover, 8 studies evaluated the 3-year survival rates of the HAR group and 5 studies compared the 5-year survival rates. The heterogeneity of both analyses was not significant (*I*^2^ = 1.0%; *I*^2^ = 0.0%). HAR was a significant poor prognostic factor for both 3-year and 5-year survival (RR = 1.57, 95% CI = 1.29–1.91, *P* < 0.001; RR = 2.55, 95% CI = 2.01–3.23, *P* < 0.001) (Fig. 3C, D).

### Publication bias and sensitivity analyses

**Fig. 4:**
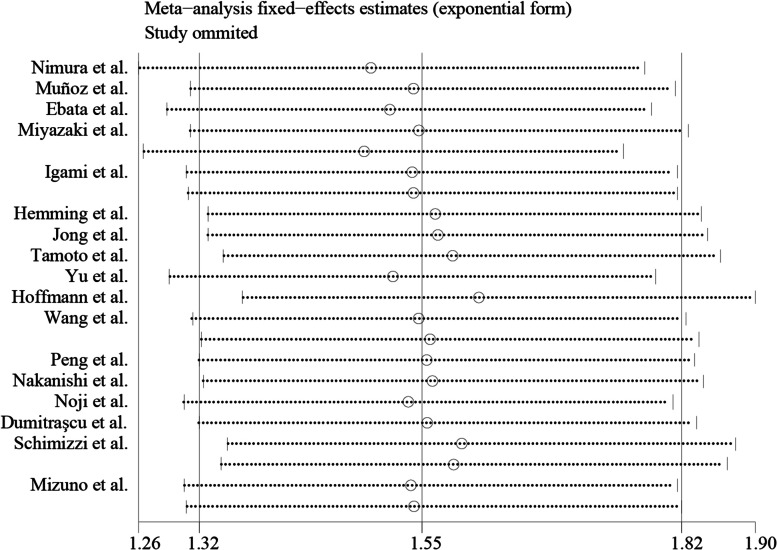
Sensitivity analyses of all studies include in this meta-analyses

The risk of publication bias was evaluated in all included studies, respectively. The results indicated that the included articles had no publication bias. Sensitivity analyses were also performed using Stata15.1 software to assess whether individual studies did not affect the overall results. The results showed that individual studies had little impact on the final results (Fig. 4).

## Discussion

This meta-analysis implies that PVR and HAR groups of Klatskin tumor have higher surgical risks than the non-vascular resection and reconstruction group. Furthermore, the vascular resection and reconstruction is a significant poor prognostic factor for poor OS. However, subgroup analyses based on the duration of the study have shown that in recent years, PVR is no longer a significant adverse prognostic factor in terms of long-term patient survival, which was further confirmed by the analyses of 3- and 5-year survival and median survival time. Besides, the HAR remains a significant adverse prognostic factor for the long-term survival of Klatskin tumor patients.

Among the studies included in this meta-analysis, most of the studies before 2010 suggested that combined vascular resection resulted in poor prognosis [9–11, 18]. In contrast, studies after 2010 mostly suggested that combined vascular resection was not associated with prognosts [13, 14, 20–22, 24, 25]. Notably, of the studies involved, Hemming et al. was the first to apply and recommend the “no-touch” resection technique of Neuhaus et al for Klatskin tumor [6, 20, 27]. Since then, this technique has been widely applied by other groups [14, 21, 23]. All of these studies showed that PVR does not lead to decreased postoperative long-term survival of patients, even though PVR tends to be performed in patients with higher tumor stage that often shows a detrimental effect on long-term survival.

The surgeon’s criteria for deciding whether to perform vascular resection during surgery also determine, to some extent, that patients receiving combined vascular resection will have relatively poorer prognosis. The decision to perform vascular resection during surgery is usually based on the following criteria: difficulty in separating the vessel from the tumor [9, 19], suspected cancer invasion of the vessel [10, 11, 15], tumor-vascular adjacency shown by preoperative imaging [14, 15, 22, 24], and intraoperative exploration revealing vascular invasion [15, 22]. However, several studies reported that the microscopic invasion of the resected portal vein does not appear to affect survival [9, 14, 21]. Also, it was mentioned in the involved studies that the vascular resection group showed more aggressive pathologic characteristics than the group without vascular resection, including tumor stage, size, liver infiltration, lymph node metastasis, and histological differentiation [7, 9, 10, 12–14, 23, 24, 26]. This suggests that the poorer prognosis of the vascular resection group may also stem from the higher malignancy of the tumors, in addition to the combined vascular resection. This can be supported by the fact that PVR and HAR tend not to be factors contributing to reduced long-term survival when the tumor stage is matched between the vascular resection group and the control group [7, 22–26]. Although Mizuno's study showed a significant decrease in long-term survival in the PVR and HAR group compared to the control group, the 3- and 5-year survival rates of the vascular resection patients with early-stage tumors were similar to those of the control group, suggesting that the heterogeneity of their study may stem from the significant difference in oncologic staging between the two groups [26].

It is noteworthy that Mayazaki’s study showed that the presence of histologically positive invasion to the portal vein had no impact on survival [10], and in most studies involving the “no-touch” resection, long-term survival in the PVR group was not significantly different from that of the control group, even though there were significant differences in the tumor stage and the extent of mobility of the portal vein between the vascular and non-vascular resection groups [14, 20, 21]. Therefore, the tumor malignancy is not the only factor affecting the long-term survival of combined vascular resection, and advanced surgical techniques can also improve the long-term survival of patients. The results of the meta-analysis also suggest that PVR no longer causes a significant decrease in the 3- and 5-year survival rates as well as the median survival time of Klatskin tumor patients.

It is also worth noting that in all known four studies, Klatskin tumor patients with combined vascular resection had a better prognosis than those unresected or unresectable cases [9–11, 18]. This also suggests that vascular resection can be performed in some Klatskin tumor patients if necessary, which reduces the likelihood of continued tumor cell spread.

According to our analyses, HAR remains a significant poor prognostic factor compared to the non-vascular resection group, and most studies on HAR suggest that HAR is associated with shorter long-term survival of Klatskin tumor patients [10, 11, 15, 26]. However, there are several studies reported different results [13, 24]. For example, in Noji’s study, the HAR group was matched one-to-one with the control group, and no significant difference in OS was observed between the two groups [24]. In Scimizzi’s study, HAR was also not a risk prognositic factor. However, the HAR group was younger and has higher ratio of neoadjuvant therapy compared with the control group in their study, which may be a major source of heterogeneity in this meta-analysis [13].

Several studies reported that adjuvant or neoadjuvant therapy may also affect the prognosis of Klatskin tumor patient. Kato et al. demonstrated that preoperative chemotherapy could shrink locally advanced cholangiocarcinoma and made it resectable, leading to significantly longer survival time [28]. Postoperative chemotherapy was also reported to be associated with favorable OS in patients with recurrent Klatskin tumors [26]. Benson et al. noted that adjuvant radiotherapy could prolong survival in patients with Klatskin tumor [29]. However, other groups reported that neoadjuvant/adjuvant radiotherapy and chemotherapy did not have a significant effect on patients’ survival [21, 30, 31]. Due to limited amount literature, small number of comparison cases, and the different conclusions between these studies, we did not discuss the potential effects of neoadjuvant/adjuvant treatments for Klatskin tumor in this meta-analysis.

Several meta-analysis papers have evaluated the effects of vascular resection on the prognosis of Klatskin tumor patients [12, 32–34]. However, these studies has several limitations. For example，only PVR was included as the type of vascular resection in the meta-analysis by Wu et al. or Chen et al. [32, 33]. HAR, also a common vascular resection modality in addition to PVR, was not included to explore its effect and compare the prognosis in the two vascular resection groups. Yu et al. systematically elaborated the effect of vascular resection on the prognosis of Klatskin tumor patients in a retrospective study and a meta-analysis in 2014 [12]. Because some studies included in this meta-analysis did not specify the type of vascular resection, more possible confounding factors may be introduced when assessing the impact of combined vascular resection on patients’ survival.

## Limitation

As mentioned above, in some studies included in this meta-analysis, there were differences in factors such as tumor stage, lymph node metastasis, and tumor size between the vascular resection group and the control group, which may caused selection bias. Besides, because the included studies were all hospital-based retrospective studies, and there were differences in the scope of included studies, surgical methods, postoperative treatment, ethnic differences, and medical institutions, which inevitably have a certain impact on the final results. The adaptability of the study results also has some limitations due to these factors. For example, the results of this study may not apply to regions that are inconsistent with the countries and ethnicities included in this study, and the differences between the experimental group and the control group in the retrospective studies will also affect the results to some degree.

## Conclusion

In general, combined vascular resection is a significant adverse prognostic factor for Klatskin tumor. With advances in surgical techniques and experience, PVR could be used when necessary to achieve R0 resection of Klatskin tumor and improve the long-term survival of patients. Whether HAR should be performed or not is still need to be considered when performing surgeries, and more well-designed clinical studies are needed to confirm the impact of HAR on prognosis in the future.

## Data Availability

All data generated or analyzed during this study are included in this published article.

## Abbreviations

PVR: Portal vein resection
HAR: Hepatic artery resection
HR: Hazard ratios
CI: Confidence interval
OS: Overall survival
RFS: Recurrence-free survival
DFS: Disease-free survival
NOS: The Newcastle-Ottawa Scale
RR: Relative risk
